# Leprosy: an ancient infectious disease with unresolved complications

**DOI:** 10.1097/JS9.0000000000000127

**Published:** 2023-01-12

**Authors:** Hitesh Chopra, Talha B. Emran, Kuldeep Dhama

**Affiliations:** a Chitkara College of Pharmacy, Chitkara University, Rajpura, Punjab; b Division of Pathology, ICAR-Indian Veterinary Research Institute, Bareilly, Izatnagar, Uttar Pradesh, India; c Department of Pharmacy, Faculty of Allied Health Sciences, Daffodil International University; d Department of Pharmacy, BGC Trust University Bangladesh, Dhaka, Bangladesh

Infected individuals with leprosy have bacteria in their bodies called *Mycobacterium leprae*, which spreads at a sluggish rate and is responsible for the disease. This condition can cause problems with the nervous system, skin, eyes, and even the nasal lining (nasal mucosa). The condition is curable if caught and treated early on. Leprosy’s patients are able to work and participate fully in society while receiving therapy. It was originally thought that leprosy was easily disseminated and deadly, but today it is curable. But if the nerve damage is not fixed, it can cause permanent problems like paralysis, blindness, and trouble moving the hands and feet^1^.

It is likely that armadillos in the south of the United States are naturally infected with the bacteria that cause leprosy in humans^2^. Though there is a little chance of contracting leprosy through interacting with armadillos, the likelihood of doing so is extremely low.

In a recent study, published in Cell Reports Medicine on November 15, 2022, researchers found that the bacteria that cause leprosy may reprogram cells in an adult animal to grow the size of the liver without producing any harm, scarring, or tumors^3^. Insights into the study’s underlying mechanisms may 1 day be used to regenerate damaged livers or rejuvenate aging livers, eliminating the need for liver transplantation.

The scarring and malignancies that ensued from earlier attempts to rebuild livers in mice using stem cells or progenitor cells were a major reason for their failure. Anura Rambukkana, a professor of regenerative medicine at the University of Edinburgh in the UK, and his colleagues found that *M. leprae* could change cells in some ways. They used this information to stop these bad things from happening.

Rambukkana and his colleagues infected nine-banded armadillos in Baton Rouge, Louisiana, in collaboration with the United States Department of Health and Human Services, and compared the livers of infected, uninfected, and resistant armadillos. The liver size of armadillos infected with *M. leprae* was found to be significantly higher compared to uninfected armadillos^3^. It is important to note that the livers of infected armadillos grew in size but otherwise seemed normal in terms of tissue architecture. The study showed that infected adult liver cells undergo partial reprogramming *in vivo*, returning to an immature condition that allows them to proliferate and regenerate. The liver grows properly as a result of the bacteria-induced regeneration, without the development of fibrotic scar tissue from repeated injury and repair or malignant cells. Scientists think that the parasite increases the number of cells inside which it can grow and replicate by reprogramming adult liver cells into a regenerative state. Similarities were discovered when the gene expression profiles of *M. leprae*-infected liver cells were compared to those of young animals and human fetal livers. This provided further evidence that *M. leprae*-infected liver cells had really achieved a rejuvenated state. Infected liver cells with *M. leprae* showed gene expression patterns that were similar to those of livers from young animals and human fetuses. There was a lot of expression of genes that control metabolism, growth, and cell division, but there was not much expression of genes that control aging. This means that the bacteria change how the liver cells control gene expression, putting them back in a progenitor state from which new liver tissue can grow.

As it is, liver disease is responsible for two million deaths annually throughout the world. The research group is hoping that their findings may lead to new treatments for liver damage and aging. However, before using this method, another question remained: how did these bacteria not kill liver cells but instead cause them to divide and reproduce? Moreover, its clinical translation to humans still needs to be verified before making it a super-glorified discovery.

## Ethical approval

Not applicable.

## Sources of funding

No funding.

## Author contributions

H.C.: conceptualization, data curation, writing – original draft preparation, writing – reviewing and editing. T.B.E.: writing – reviewing and editing, visualization, supervision. K.D.: data curation, writing – original draft preparation, writing – reviewing and editing.

## Conflicts of interest disclosure

The authors declare that they have no financial conflict of interest with regard to the content of this report.

## Research registration unique identifying number

Not applicable.

## Guarantor

Talha Bin Emran, PhD.

## Provenance and peer review

Not commissioned, internally peer-reviewed.

## Data statement

No specific data collected for the above manuscript.
